# Integrating *Trissolcus japonicus* (Ashmead, 1904) (Hymenoptera: Scelionidae) into Management Programs for *Halyomorpha halys* (Stål, 1855) (Hemiptera: Pentatomidae) in Apple Orchards: Impact of Insecticide Applications and Spray Patterns

**DOI:** 10.3390/insects11120833

**Published:** 2020-11-26

**Authors:** Dalton C. Ludwick, Jessica Patterson, Layne B. Leake, Lee Carper, Tracy C. Leskey

**Affiliations:** 1 Appalachian Fruit Research Station, USDA-ARS, Kearneysville, WV 25430, USA; Lee.Carper@usda.gov (L.C.); Tracy.Leskey@usda.gov (T.C.L.); 2 Department of Entomology, Texas A&M AgriLife Research & Extension Center, 10345 Highway 44, Corpus Christi, TX 78406, USA; 3 Institute of Environment and Physical Sciences, Shepherd University, Shepherdstown, WV 25443, USA; jpatte03@rams.shepherd.edu; 4 Division of Plant Sciences, University of Missouri-Columbia, Columbia, MO 65211, USA; lblc8c@mail.missouri.edu

**Keywords:** parasitoids, chemical control, tree fruit

## Abstract

**Simple Summary:**

In the United States, the invasive brown marmorated stink bug has caused economic damage to specialty crop and tree fruit industries, including apples. Since its arrival, research has shown that altered spray programs can control brown marmorated stink bug as well as spraying an entire apple orchard, resulting in less insecticide use. Here, we evaluated three spray programs and four commonly used insecticides in combination for their impact on a non-native wasp, the samurai wasp, that parasitizes egg masses of this invasive stink bug. We exposed adult samurai wasps to egg masses from our treatments, including unsprayed areas, and recorded their survival as well as how many wasps emerged afterward. In addition, we recorded how immature wasps developing inside egg masses responded to these treatments. We found that only one insecticide had negative impacts on the adult wasps while the majority of insecticides tested impacted their offspring’s emergence. In general, insecticides tested minimally impacted wasps already developing inside egg masses treated with insecticides. These data support the idea that growers can simultaneously manage brown marmorated stink bug and conserve beneficial insects, such as the samurai wasp, while reducing insecticide use by using improved pest management tactics.

**Abstract:**

*Halyomorpha halys* (Stål, 1855) (Hemiptera: Pentatomidae) is an invasive species in the United States, where it has caused significant damage to specialty crops, including apples. While integrated pest management techniques have been developed for *H. halys* in apple, including spray application techniques, it is unknown how these techniques affect foraging, adventive *Trissolcus japonicus* (Ashmead, 1904) (Hymenoptera: Scelionidae), and its offspring. In this study, egg masses (unparasitized and 2 and 7 day parasitized pre-treatment) were placed in apple orchards in treated and untreated locations that received full block insecticide applications or reduced application techniques, including border row or alternate row middle applications. Bifenthrin, thiamethoxam + λ-cyhalothrin, clothianidin, and methomyl were evaluated. Egg masses were retrieved 24 h after spray applications. For 2 and 7 day parasitized pre-treatment, adult *T. japonicus* emergence was recorded from each egg mass. For unparasitized egg masses, *T. japonicus* females were given 24 h to forage and oviposit on post-treatment egg masses with female survivorship, and adult emergence from egg masses was recorded. Female survivorship was significantly lower on post-treatment egg masses retrieved from areas receiving bifenthrin applications. Emergence from post-treatment egg masses was affected by thiamethoxam + λ-cyhalothrin, bifenthrin, and methomyl in some treated areas, whereas less impact was observed on 2 and 7 day pre-treatment parasitized egg masses in general. These data provide further insights into *H. halys* management and the potential impact of *T. japonicus* in sprayed orchard agroecosystems.

## 1. Introduction

*Halyomorpha halys* (Stål, 1855) (Hemiptera: Pentatomidae) likely invaded the United States of America (USA) in the 1990s, although the first specimen was not identified until 2001 [1]. Since then, *H. halys* populations have grown and become economically damaging in agricultural systems, particularly in specialty and row crops [2]. In addition, *H. halys* is a nuisance pest because of its fall dispersal to and overwintering within human-made structures [3,4,5,6]. Additionally, *H. halys* has invaded other countries and continents, some of which are now experiencing serious crop damage and nuisance problems [2,7,8,9].

While insecticide applications have been used to mitigate the risk of injury by *H. halys*, biological control is considered the most promising long-term strategy for its management. However, the severity of damage inflicted by *H. halys* is believed to be due in part due to its release from co-evolved natural enemies in its native range [10]. Biological control from native predators and parasitoids in the invaded range [11] has been inadequate to suppress *H. halys* populations, at least in the short term. In its native range, *H. halys* populations are believed to be reduced substantially by *Trissolcus japonicus* (Ashmead, 1904) (Hymenoptera: Scelionidae), an egg parasitoid [12,13], although recent models indicate their impact on populations could be somewhat overestimated [14]. However, with continued population pressure from *H. halys* in invaded regions, researchers in the USA have been evaluating its host specificity in quarantine facilities in anticipation of future classical biological programs [13,15,16]. Additionally, adventive populations of the *T. japonicus* have been discovered in more than a dozen states since 2014 [17,18,19,20,21,22,23]. Redistribution efforts have occurred within some of these states to enhance its establishment and increase its geographic range [19,24]

*Trissolcus japonicus* was originally recovered from wooded and non-agricultural areas [17,18,19,20,21,22,23], but recent detections have included agricultural sites [20,25]. While these findings are promising for long-term biological control efforts, we do not know how particular management programs for specific crops may affect *T. japonicus* establishment and impact on *H. halys* populations. In apple orchards, *H. halys* has been managed with broad-spectrum insecticides [26,27] applied to the entire orchard or to select areas of the orchard (e.g., border sprays) [28,29] in order to provide refugia for natural enemies. However, we do not know how some of these more commonly applied materials used under various management scenarios may affect adult survivorship and offspring emergence from parasitized egg masses. To address this gap in knowledge, we evaluated four insecticides and three spray patterns commonly used for *H. halys* management in apple orchards for their impact on adult *T. japonicus* foraging on exposed egg masses following a spray application and emergence of their offspring, and on offspring developing inside egg masses parasitized prior to insecticide treatment.

## 2. Materials and Methods

### 2.1. Field Plots

Orchard blocks used in this study were maintained at the USDA-ARS Appalachian Fruit Research Station in Kearneysville, WV, according to standard horticultural practices and pest management practices for major insect pests such as *Conotrachelus nenuphar* (Herbst, 1797) (Coleoptera: Curculionidae), *Cydia pomonella* (Linnaeus, 1758) (Lepidoptera: Tortricidae), and *Grapholita molesta* (Busck, 1916) (Lepidoptera: Tortricidae), and plant diseases [30]. Materials selected for routine block maintenance were restricted to compounds known to be minimally lethal to *H. halys* and were applied via an air-blast sprayer with water at 935 L/ha. Additional horticultural information can be found in Table 1.

### 2.2. Halyomorpha Halys Colony

Insects for this trial originated from the New Jersey Department of Agriculture and were maintained at the Appalachian Fruit Research Station, as described in Ludwick et al. (submitted). Briefly, cages with ~50 adults were provisioned with a potted green bean plant (*Phaseolus vulgaris* L.), potted *Peperomia* sp., and water via dental wicks (Absorbal, Wheat Ridge, CO, USA) in 50 mL Erlenmeyer flasks changed weekly. Egg masses were collected from the adult cages three times per week for colony maintenance. *Halyomorpha halys* colony cages were maintained in a room at 25 °C, with a 16:8 (L:D) photoperiod and 50–70% relative humidity. Egg masses were either used as a source of nymphs to maintain the *H. halys* colony or were stored at −80 °C (Model ULT790-3-D31, Kendro Laboratory Products, Asheville, NC, USA) until needed for *T. japonicus* colony maintenance or experimentation.

### 2.3. Trissolcus Japonicus Colony

The *T. japonicus* colony, as described in Ludwick et al. [31], originated from field-collected specimens in West Virginia. Adult wasps were provided honey and were maintained under the same conditions as the *H. halys* colony. Adults were placed in 7-dram vials with thawed egg masses on card stock for 24–72 h to produce new wasps. Egg masses were held in place with double-sided tape that had exposed areas covered with fine sand (BE Good Company, San Mateo, CA, USA). Approximately 15 egg masses were exposed to adult female wasps per week to maintain a colony of ~1200 individuals.

### 2.4. Egg Mass Deployment

Experiments in 2018 were conducted using fresh egg masses (<24 h) and egg masses frozen for less than 10 weeks, while those in 2019 were solely from frozen egg masses (<8 weeks). All egg masses were placed on card stock as done with the *T. japonicus* colony described above.

In 2018, egg masses parasitized 6–7 days prior to, and egg masses parasitized just after insecticide applications, referred to as 7 day pre-treatment and post-treatment egg masses, respectively, were included. In 2019, egg masses parasitized 2 days prior to insecticide applications and referred to as 2-day pre-treatment were also included. All egg masses were affixed to the undersides of leaves with No. 2 insect pins near the edge of the canopy dripline at ~1.7 m in height in a border row or interior (14.7 m from border) apple tree or on a wild host tree just outside the orchard block (9.8 m from orchard border). Depending on egg mass availability, two to four trees were used in each location for all egg mass treatments present on each tree in 2018 and 2019.

### 2.5. Insecticide Applications

In 2018, insecticides included in the study were bifenthrin (Brigade WSB, FMC, Philadelphia, PA, USA; at 1.12 kg/ha) and clothianidin (Belay, Valent, Walnut Creek, CA, USA) at 0.44 L/ha; in 2019, methomyl (Lannate SP, DuPont, Wilmington, DE, USA) at 1.12 kg/ha and thiamethoxam + λ-cyhalothrin (Endigo ZC, Syngenta, Greensboro, NC, USA) at 0.44 L/ha were added, with all insecticides applied at 935.4 L of water/ha. For border applications along the length of a row, egg masses were deployed in trees in the sprayed border and unsprayed interior rows (14.7 m from orchard border); this was the only application method evaluated in 2018. In 2019, in addition to border spray applications, we incorporated alternate row middle (A.R.M.) sprays as described in Short et al. (2017), in which every other row is sprayed and a complete block spray in which every row is sprayed. In the A.R.M. sprays, egg masses were placed in the sprayed border row and an unsprayed gap row (5.5 m from orchard border). In the complete block sprays, egg masses were deployed in trees in the sprayed border and interior rows (5.5 m from orchard border). Additionally, egg masses were deployed in unsprayed wild host trees in wooded treelines adjacent to blocks that received border row or complete block sprays; these egg masses served as a control. Each block used in 2019 was assigned one of the three spray patterns for the field season and the same insecticide was applied to all blocks on the same date (Table 1).

Once all egg masses were deployed, blocks were sprayed with an insecticide and egg masses were collected 24 h later. If card stock were damp at the time of collection, a fume hood was used for up to 6 h to dry them to prevent fungal growth. For so-called post-treatment egg masses, each were then exposed to a single female *T. japonicus* for 24 h; the fate of the wasp was recorded as alive, moribund, or dead. All pre- and post-treatment egg masses were evaluated after 4 weeks to determine the number of wasps that had emerged and the number of remaining eggs.

### 2.6. Statistical Analyses

Data were analyzed in SAS version 9.4 (SAS Institute, Cary, NC, USA). In this study, each egg mass was considered an individual replicate egg mass availability and replicates were accumulated through time in 2018 and in 2019. The proportion of an egg mass that emerged was calculated by dividing the number of emerged adults by the total number of eggs. Adult female survivorship was calculated by dividing the number of alive after 24 h by the total number of females exposed to non-parasitized egg masses. Data were analyzed separately for each year and for female survivorship as well as egg mass emergence for each insecticide treatment; data were arcsine square-root transformed and analyzed with analysis of variance (ANOVA) (PROC GLM) in SAS version 9.4 (Table 2). If the *F*-test was significant, then post hoc pairwise comparisons were made for female survivorship on and emergence from egg masses deployed in the untreated woodline (control), and all other treatments were calculated using Dunnett’s test.

## 3. Results

### 3.1. 2018

**Figure 1:**
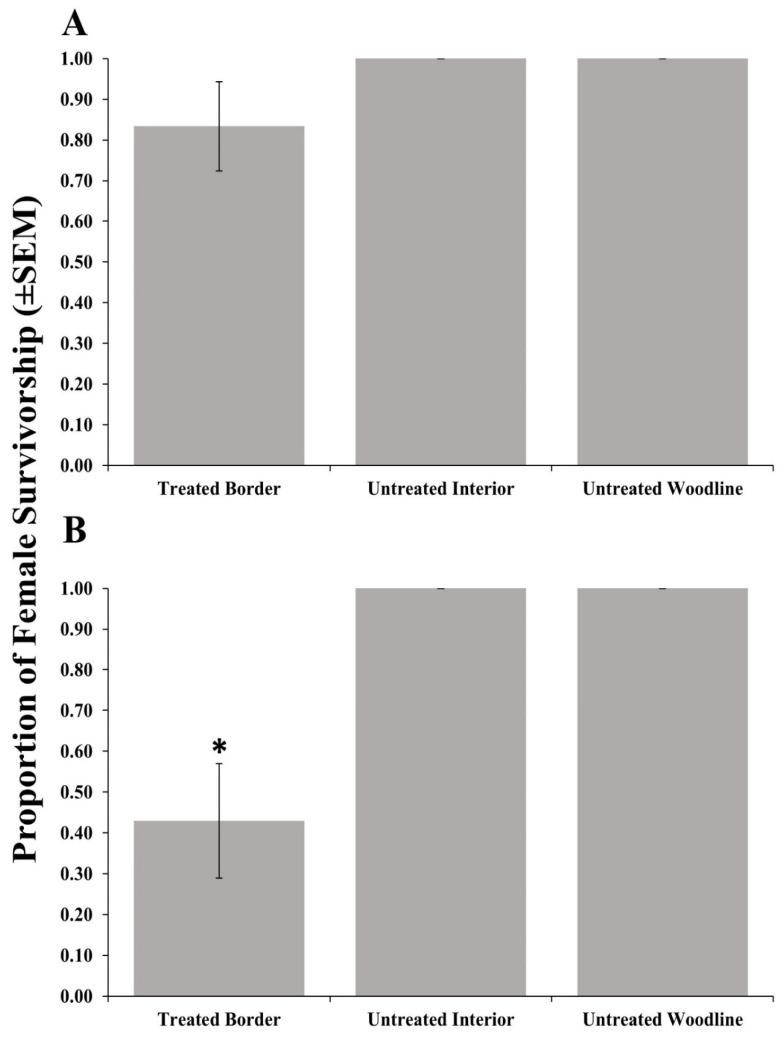
Survivorship (±SEM) of female *Trissolcus japonicus* provided egg masses retrieved from three apple orchard locations (border row, interior row, and untreated woodline) where the border row was treated with clothianidin (**A**) or bifenthrin (**B**) in 2018. Pairwise comparisons of female survivorship provided egg masses retrieved from the untreated woodline (control), and other egg mass deployment locations were calculated via a Dunnett’s test (*p* < 0.05); significant differences are indicated with an asterisk.

Adult female survivorship after foraging on (Figure 1A) post-treatment egg masses recovered from border areas treated with clothianidin and subsequent progeny emergence (Table 3) from those egg masses was not significantly different from post-treatment egg masses placed in the untreated woodline (Table 2). Conversely, female survivorship (Figure 1B) from post-treatment egg masses recovered from border areas treated with bifenthrin and progeny emergence (Table 4) was significantly reduced compared with those egg masses recovered from the untreated woodline. No negative effects on survivorship or progeny emergence were detected from post-treatment egg masses deployed in the unsprayed interior row for either material. Interestingly, emergence from 7 days pre-treatment parasitized egg masses deployed in orchard borders treated with clothianidin and bifenthrin were not significantly different from those deployed in the untreated interior rows or woodline (Table 2, Table 3 and Table 4).

### 3.2. 2019

**Figure 2:**
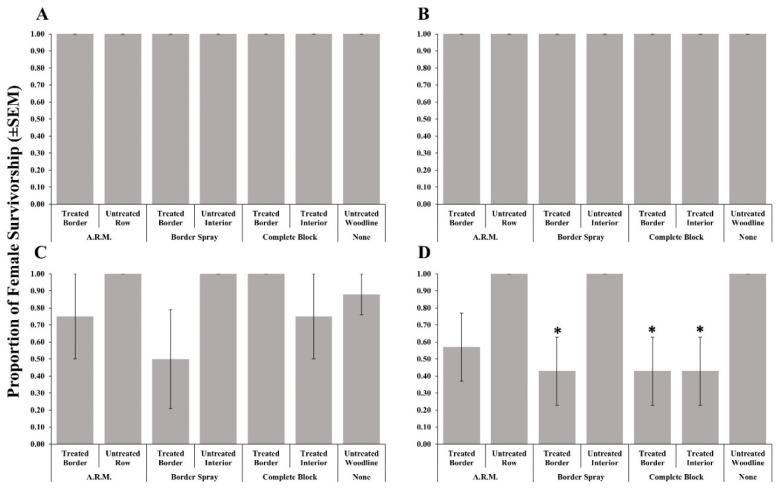
Survivorship (±SEM) of female *Trissolcus japonicus* provided egg masses retrieved from three apple orchard locations where a border row, alternate row middle (A.R.M.), or complete block spray pattern. Materials evaluated included methomyl (**A**), thiamethoxam + λ-cyhalothrin (**B**), clothianidin (**C**), or bifenthrin (**D**) in 2019. Pairwise comparisons of female survivorship provided egg masses retrieved from the untreated woodline (control), and other egg mass deployment locations were calculated via a Dunnett’s test (*p* < 0.05); significant differences are indicated with an asterisk.

Adult female survivorship after foraging on post-treatment egg masses recovered from treated rows (border, complete, and A.R.M. applications) was not affected by methomyl (Table 2, Figure 2A), thiamethoxam + λ-cyhalothrin (Figure 2B), or clothianidin (Figure 2C) applications. As in 2018, bifenthrin applications resulted in significantly reduced female survivorship when given post-treatment egg masses recovered from the treated border row from border spray applications, and the treated border and interior rows from the complete block application when compared to the untreated woodline (Figure 2D).

Reductions in adult emergence from post-treatment parasitized egg masses in the following scenarios when compared to the respective untreated woodlines: treated borders of complete block applications of methomyl (Table 5), nearly all locations treated with thiamethoxam + λ-cyhalothrin (Table 6), and from those recovered from border and interior rows treated with complete block bifenthrin applications (Table 7). For pre-treatment egg masses parasitized 2 days and 6–7 days prior to deployment, no significant differences in emergence were detected from those deployed in trees treated and untreated orchard rows when compared with emergence from egg masses deployed in the untreated woodline (Table 5, Table 6, Table 7 and Table 8), similar to what was observed in 2018.

## 4. Discussion

*Halyomorpha halys* has been managed primarily through insecticide applications [2,26,32,33]. Research on its behavior and ecology has facilitated the development and implementation of integrated pest management (IPM) strategies [29,34], including attract-and-kill [35], border spray [28,36], and threshold-based spray [37] strategies. However, these strategies only serve to manage *H. halys* in treated crops, leaving unmanaged areas to act as reservoirs for re-infestation. Landscape level solutions, such as classical biological control with *T. japonicus*, are needed to reduce *H. halys* populations, thereby reducing the need for insecticide applications. However, in all likelihood, *T. japonicus* alone may not be able to reduce populations to a level that mitigates the need for additional intervention and/or other biological control agents [14]; thus, understanding how insecticides and specific IPM tactics utilizing them affect *T. japonicus* development and survivorship are critical for to enabling growers to make informed decisions. 

Indeed, adult parasitoid exposure to host egg masses treated with an insecticide can significantly reduce parasitism and adult survivorship [38,39,40]. In our study, female *T. japonicus* survivorship was most affected when provided with post-treatment egg masses exposed to bifenthrin applications, indicating that this material could reduce *T. japonicus* foraging success in treated crops. Moreover, Lowenstein et al. [41] also found that this insecticide resulted in high mortality of adult females if directly exposed to the spray material. Bifenthrin itself, while considered one of the most effective materials against *H. halys* [27,42], is only labelled for limited emergency use exemptions in tree fruit in a few states in the USA. Thus, foraging *T. japonicus* populations may not frequently encounter this material in sprayed orchards, although other materials such as thiamethoxam also induced high mortality when adult females were directly exposed to this material as well [41]. In our study, mortality of adult females foraging on exposed egg masses to other insecticide applications was also reduced, although not significantly, in some cases by clothianidin, but not for methomyl or thiamethoxam + λ-cyhalothrin. Many insecticides commonly used for *H. halys* control have short residual activity on this pest [42] and possibly foraging *T. japonicus* adults.

For progeny generated by foraging females from post-treatment egg masses, there were significant reductions in adult emergence from egg masses recovered from some treated areas sprayed with methomyl, thiamethoxam + λ-cyhalothrin, and bifenthrin; however, this trend was not consistent. In all likelihood, *T. japonicus* progeny were affected inconsistently due to the variation in tree architecture among cultivars used in this study. In some cases, eggs were somewhat shielded from insecticide applications. In hindsight, the deployment of spray cards would have been useful in deciphering these data and would be recommended for any similar future studies. However, it does seem likely that *T. japonicus* can survive and develop in sprayed crops to some degree as egg masses yielding *T. japonicus* have been recovered in other studies in treated crops, including peach [20] and apple [25].

Parasitized egg masses exposed to insecticides also can result in reduced emergence [39,40,43]. In some instances, developing parasitoids vary in susceptibility to insecticides depending on the age of the insect at time of exposure [44]. However, developing parasitoids are not always susceptible to insecticides, as developing *Trissolcus basalis* were not sensitive to permethrin or methyl parathion [38]. In our study, we exposed egg masses parasitized 2 and 7 days prior to deployment in treated and untreated areas with three different spray patterns and four insecticides, finding no significant impacts on emergence when compared with emergence from egg masses deployed in an unsprayed wooded treeline. Thus, it appears that under these conditions, *T. japonicus* can complete development in egg masses present in treated areas. These results indicate that this biological control agent can contribute to the management of *H. halys* under these circumstances, but that overall biological control impacts likely will be maximized by reducing insecticide inputs.

*Trissolcus japonicus* has largely been reported from urban or unmanaged wooded environments [14,17,18,22,45], but it has also been found in peach and apple orchards [20,25]. On the basis of our results, however, both adult survivorship and subsequent adult emergence in orchards was greatest in untreated refugia created by IPM tactics such as border or A.R.M. sprays. Thus, to maximize the impact of *T. japonicus*, using IPM tactics such as trap-based treatment thresholds [37], border sprays [28,36], or attract-and-kill [29,35] that provide either reductions in treated areas or number of applications would likely increase *T. japonicus* foraging. In addition, we recommend growers consider use of methomyl, thiamethoxam + λ-cyhalothrin, and clothianidin due to the minimal impacts on foraging and developing *T. japonicus*. These tactics also obviously reduce insecticide input and prevent secondary pest outbreaks. Ultimately, to manage *H. halys* effectively and sustainably, we must consider ways to integrate these factors into managed areas.

## 5. Conclusions

1. Identified that the insecticide bifenthrin had consistent negative impacts on *Trissolcus japonicus*.
2. Insecticide applicationss did not significantly impair *Trissolcus japonicus* emergence from egg masses parasitized prior to spray applications.
3. Management of *Halyomorpha halys* in apple orchards is amenable to adjustments that support the incorporation of *Trissolcus japonicus*. These data on combining insecticides and biological control agents may aid in the reduction of *Halyomorpha halys* populations across orchard agroecosystems.

## Figures and Tables

**Table 1:** Horticultural information for blocks used in this study in 2018 and 2019.

Spray Pattern	Cultivar	Plant Date	Area (ha)	Tree Spacing (m × m)
Border row	Granny Smith, Royal Empire, Standard Delicious, Golden Delicious, Goldspur, Spur Red	2008	0.11	4.9 × 4.9
Alternate row middle	Gala	1998	0.15	1.5 × 5.5
complete block	Ida Red	2006	0.06	2.4 × 4.9

**Table 2:** ANOVA table for 2018 and 2019 pesticide data.

Year	Variable	Insecticide	Num. D.F.	Den. D.F.	*F*-Value	*p* > *F*
2018	Female survivorship	Clothianidin	2	34	2.30	0.1159
		Bifenthrin	2	42	19.29	<0.0001
	Post-treatment parasitism emergence	Clothianidin	2	33	0.76	0.4766
		Bifenthrin	2	43	10.95	0.0001
	7 day parasitized pre-treatment emergence	Clothianidin	2	44	1.05	0.3575
		Bifenthrin	2	31	1.23	0.3062
2019	Female survivorship	Methomyl	6	45	.	.
		Thiamethoxam + λ-cyhalothrin	6	49	.	.
		Clothianidin	6	25	1.39	0.2578
		Bifenthrin	6	46	4.36	0.0015
	Post-treatment parasitism emergence	Methomyl	6	46	2.97	0.0156
		Thiamethoxam + λ-cyhalothrin	6	49	5.73	0.0001
		Clothianidin	6	44	0.79	0.5860
		Bifenthrin	6	48	3.63	0.0047
	2 day parasitized pre-treatment emergence	Methomyl	6	37	1.15	0.3511
		Thiamethoxam + λ-cyhalothrin	6	40	1.06	0.3998
		Clothianidin	6	39	1.08	0.3883
		Bifenthrin	6	40	0.49	0.8132
	7 day parasitized pre-treatment emergence	Methomyl	6	36	1.76	0.1348
		Thiamethoxam + λ-cyhalothrin	6	41	1.67	0.1528
		Clothianidin	6	36	2.24	0.0617
		Bifenthrin	6	40	0.36	0.9014

**Table 3:** Emergence (±SEM) of *Trissolcus japonicus* from egg masses placed at three locations in an apple orchard treated with clothianidin. The number of replicates for each treatment is presented in parentheses.

Location	Post-Treatment Parasitized	7 Day Parasitized Pre-Treatment
Treated border	0.33 ± 0.10 (12)	0.81 ± 0.09 (16)
Untreated interior	0.56 ± 0.11 (13)	0.90 ± 0.06 (16)
Untreated woodline	0.50 ± 0.12 (11)	0.62 ± 0.10 (16)

**Table 4:** Emergence (±SEM) of *Trissolcus japonicus* from egg masses placed at three locations in an apple orchard treated with bifenthrin. The number of replicates for each treatment is presented in parentheses. Values with an asterisk are significantly different (*p* < 0.05) from the untreated woodline value in that column.

Location	Post-Treatment Parasitized	7 Day Parasitized Pre-Treatment
Treated border	0.15 ± 0.09 (15) *	0.70 ± 0.11 (11)
Untreated interior	0.66 ± 0.09 (15)	0.55 ± 0.10 (12)
Untreated woodline	0.64 ± 0.09 (16)	0.78 ± 0.08 (12)

**Table 5:** Emergence (±SEM) of *Trissolcus japonicus* from egg masses placed at three locations in an apple orchard treated with methomyl. The number of replicates for each treatment is presented in parentheses. Values with an asterisk are significantly different (*p* < 0.05) from the untreated woodline value in that column.

Spray Pattern	Location	Post-Treatment Parasitized	2 Day Parasitized Pre-Treatment	7 Day Parasitized Pre-Treatment
A.R.M.	Treated border	0.78 ± 0.06 (8)	0.36 ± 0.16 (3)	0.29 ± 0.18 (3)
	Untreated row	0.75 ± 0.11 (9)	0.76 ± 0.10 (7)	0.57 ± 0.15 (7)
Border spray	Treated border	0.94 ± 0.03 (7)	0.37 ± 0.23 (3)	0.03 ± 0.01 (3)
	Untreated interior	0.69 ± 0.10 (7)	0.54 ± 0.16 (7)	0.61 ± 0.14 (7)
Complete block	Treated border	0.32 ± 0.14 (5) *	0.69 ± 0.16 (3)	0.15 ± 0.14 (3)
	Treated interior	0.60 ± 0.11 (7)	0.67 ± 0.15 (7)	0.39 ± 0.13 (7)
N/A	Untreated woodline	0.75 ± 0.09 (14)	0.74 ± 0.08 (14)	0.51 ± 0.09 (14)

**Table 6:** Emergence (±SEM) of *Trissolcus japonicus* from egg masses placed at three locations in an apple orchard treated with thiamethoxam + λ-cyhalothrin. The number of replicates for each treatment is presented in parentheses. Values with an asterisk are significantly different (*p* < 0.05) from the untreated woodline value in that column.

Spray Pattern	Location	Post-Treatment Parasitized	2 Day Parasitized Pre-Treatment	7 Day Parasitized Pre-Treatment
A.R.M.	Treated border	0.36 ± 0.14 (7)	0.10 ± 0.05 (6)	0.54 ± 0.11 (6)
	Untreated row	0.26 ± 0.12 (7) *	0.49 ± 0.11 (6)	0.55 ± 0.17 (6)
Border spray	Treated border	0.21 ± 0.04 (7) *	0.40 ± 0.18 (6)	0.26 ± 0.15 (6)
	Untreated interior	0.31 ± 0.13 (7) *	0.45 ± 0.14 (6)	0.51 ± 0.14 (6)
Complete block	Treated border	0.16 ± 0.10 (7) *	0.23 ± 0.14 (6)	0.47 ± 0.13 (6)
	Treated interior	0.03 ± 0.02 (7) *	0.30 ± 0.15 (6)	0.12 ± 0.08 (6)
N/A	Untreated woodline	0.73 ± 0.09 (14)	0.27 ± 0.10 (12)	0.44 ± 0.08 (12)

**Table 7:** Emergence (±SEM) of *Trissolcus japonicus* from egg masses placed at three locations in an apple orchard treated with bifenthrin. The number of replicates for each treatment is presented in parentheses. Analyses were conducted for each column of data. Values with an asterisk are significantly different (*p* < 0.05) from the untreated woodline value in that column.

Spray Pattern	Location	Post-Treatment Parasitized	2 Day Parasitized Pre-Treatment	7 Day Parasitized Pre-Treatment
A.R.M.	Treated border	0.36 ± 0.13 (7)	0.59 ± 0.05 (6)	0.47 ± 0.16 (6)
	Untreated row	0.65 ± 0.06 (7)	0.71 ± 0.15 (6)	0.60 ± 0.14 (6)
Border spray	Treated border	0.31 ± 0.13 (7)	0.55 ± 0.13 (6)	0.49 ± 0.14 (6)
	Untreated interior	0.49 ± 0.15 (7)	0.47 ± 0.14 (6)	0.59 ± 0.17 (6)
Complete block	Treated border	0.17 ± 0.06 (7) *	0.57 ± 0.13 (6)	0.57 ± 0.11 (6)
	Treated interior	0.09 ± 0.05 (7) *	0.46 ± 0.10 (6)	0.50 ± 0.14 (6)
N/A	Untreated woodline	0.66 ± 0.10 (13)	0.59 ± 0.07 (12)	0.44 ± 0.10 (12)

**Table 8:** Emergence (±SEM) of *Trissolcus japonicus* from egg masses placed at three locations in an apple orchard treated with clothianidin. The number of replicates for each treatment is presented in parentheses. Analyses were conducted for each column of data.

Spray Pattern	Location	Post-Treatment Parasitized	2 Day Parasitized Pre-Treatment	7 Day Parasitized Pre-Treatment
A.R.M.	Treated border	0.39 ± 0.15 (7)	0.75 ± 0.17 (5)	0.18 ± 0.16 (6)
	Untreated row	0.55 ± 0.19 (5)	0.94 ± 0.02 (5)	0.70 ± 0.15 (5)
Border spray	Treated border	0.38 ± 0.15 (7)	0.78 ± 0.05 (6)	0.74 ± 0.10 (6)
	Untreated interior	0.64 ± 0.19 (6)	0.46 ± 0.21 (6)	0.71 ± 0.13 (5)
Complete block	Treated border	0.26 ± 0.16 (6)	0.57 ± 0.17 (6)	0.70 ± 0.14 (6)
	Treated interior	0.24 ± 0.10 (7)	0.59 ± 0.17 (6)	0.40 ± 0.14 (5)
N/A	Untreated woodline	0.30 ± 0.11 (13)	0.76 ± 0.09 (12)	0.59 ± 0.13 (10)
